# Annals of General Psychiatry reviewer acknowledgement 2013

**DOI:** 10.1186/1744-859X-13-3

**Published:** 2014-02-03

**Authors:** Konstantinos N Fountoulakis

**Affiliations:** 1 Department of Psychiatry, School of Medicine, Aristotle University of Thessaloniki, Thessaloniki, Pylaia 55535, Greece

## Abstract

**Contributing reviewers:**

The editors of *Annals of General Psychiatry* would like to thank all of our reviewers who have contributed to the journal in volume 12 (2013).

## 

Cengiz Akkaya

Turkey

Umberto Albert

Italy

Tadao Arinami

Japan

Jean-Michel Azorin

France

Chris Baeken

Belgium

Filipe Barbosa

Portugal

Alasdair Barr

Canada

Marianne Barton

United States of America

Jordana Bayer

Australia

Ety Berant

Israel

Francesca Brambilla

Italy

Jerome Brunelin

France

Tommaso Cai

Italy

Philip Candilis

United States of America

Andre Carvalho

Brazil

Janet Catov

United States of America

Andrea Cavanna

United Kingdom

John Chapman

United States of America

Han-Ying Chen

Taiwan

Mark Detweiler

United States of America

Carol Dillon

Argentina

Viktorija Dragojevic-Simic

Serbia

Tatiana Dubayova

Slovakia

Simon Dymond

United Kingdom

Maria Luisa Figueira

Portugal

Michele Fornaro

Italy

Soma Ganesan

Canada

Keming Gao

United States of America

Gabor Gazdag

Hungary

Mark George

United States of America

Yuri Gimelfarb

Israel

Hans Grabe

Germany

Nisara Granado

United States of America

Rolf W. Gråwe

Norway

Gülcan Güleç

Turkey

Barbara Hanusa

United States of America

Setsko Hanzawa

Japan

Magnus Haraldsson

Iceland

Egbert Hartstra

Belgium

Philip Harvey

United States of America

Kenji Hashimoto

Japan

Pia Heppner

United States of America

Harriet Hiscock

Australia

Cyril Hoschl

Czech Republic

Shinji Iizaka

Japan

Manabu Ikeda

Japan

Qin Jiang

United States of America

Christopher Kearney

United States of America

Bruce Kinon

United States of America

Taishiro Kishimoto

Japan

Alexandra Kleiman

Germany

Michael Koelch

Germany

Tsuyoshi Kondo

Japan

Jyrki Korkeila

Finland

Frank Kozel

United States of America

Itaru Kushima

Japan

Lawrence T. Lam

Hong Kong

Cynthia LeardMann

United States of America

Claude Leclerc

Canada

Stefan Leucht

Germany

Adam Lewin

United States of America

Jean-Pierre Lindenmayer

United States of America

Juan V. Luciano

Spain

Arja Mainio

Finland

Louise Marryat

United Kingdom

Almudena Martorell

Spain

Valentin Mbekou

Canada

Roumen Milev

Canada

Ali Montazeri

Iran

Lindee Morgan

United States of America

Gunnar Morken

Norway

Kai Müller

Germany

Noeline Nakasujja

Uganda

Michael Gerhard Odenwald

Germany

Takashi Okada

Japan

João Oliveira

Brazil

Juhi Pandey

United States of America

Andreas Pavalkis

Cyprus

Vassilis Pavlopoulos

Greece

Jesus Perez

United Kingdom

Bernice Pescosolido

United States of America

Antonio Preti

Italy

Javier Quintero

Spain

Rajeev Ramchand

United States of America

Keith Rasmussen

United States of America

Helen Richards

United Kingdom

Soledad Romero

Spain

Patricia Rosebush

Canada

Nicolas Rusch

United States of America

Ann-Margret Rydell

Sweden

Craig Sawchuk

United States of America

William Sledge

United States of America

Rhonda Small

Australia

Jorgen Sundby

Norway

Bruce Sutor

United States of America

Sara Tai

United Kingdom

Kei-ichiro Takase

Japan

Cornelia Thiels

Germany

M. Inmaculada Torres

Spain

Karli Treyvaud

Australia

Wataru Ukai

Japan

Carole Upshur

United States of America

Brian van Wyk

South Africa

Areti Angeliki Veroniki

Greece

Simone Vigod

Canada

Martin Voracek

Austria

Margareta Westerbotn

Sweden

Leenika Wijeratne

Sri Lanka

Allison Williams

United States of America

Janet Williams

United States of America

Andreas Wittorf

Germany

Heather Yardley

United States of America

Norio Yasui-Furukori

Japan

Reiji Yoshimura

Japan

